# Typology of Family Support in Home Care for Iranian Older People: A Qualitative Study

**DOI:** 10.3390/ijerph18126361

**Published:** 2021-06-11

**Authors:** Soheila Shamsikhani, Fazlollah Ahmadi, Anoshirvan Kazemnejad, Mojtaba Vaismoradi

**Affiliations:** 1 Faculty of Medical Sciences, Tarbiat Modares University, Tehran 14155-4838, Iran; s.shamsikhani@modares.ac.ir (S.S.); kazem_an@modares.ac.ir (A.K.); 2 Faculty of Nursing and Health Sciences, Nord University, 8049 Bodø, Norway; mojtaba.vaismoradi@nord.no

**Keywords:** support, older people, parent, expectations, home care

## Abstract

The world population is rapidly aging. In older people, age-related biological decline in most body systems causes functional decline, an increase in dependence, and an increased need for support, especially by their family members. The aim of this study was to explore the main aspects of family support for older parents in home care. This qualitative study was conducted using a deductive qualitative content analysis approach. Participants were 21 older parents living in their own homes, as well as four family members of some participants. Data were collected using semi-structured interviews and then were analyzed using the primary matrix developed based on the existing literature. The main aspects of family support for older parents were grouped into five predetermined categories and one new category: “instrumental support”, “financial support”, “psycho-emotional support”, “healthcare-related support”, “informational-technological support”, and “social preference support “. Family support for older people in home care is a multi-dimensional phenomenon. Family members can identify the needs of their older parents and provide them with appropriate support in collaboration with healthcare professionals to enhance their quality of life, autonomy, and satisfaction with life.

## 1. Introduction

The world population is rapidly aging, and life expectancy has reached more than sixty years [1]. The population of older people in Iran is also progressively increasing. Accordingly, Iran has become the second country with respect to the growth rate of the older population across the globe. Estimates predict that the population of people aged ≥60 years in Iran will reach 29 million by 2050, though the total population of Iran will be 92 million [2].

As a dynamic and complex natural process, aging negatively influences the physical, mental, and social status of people [3]. It causes a functional decline in most body systems [4] and thereby leads to different health-related problems [5]. Age-related functional decline and health-related problems in this age group increase their dependency on others [4]. Their needs vary based on their age, education level, gender, living status, and occupational background [6], and they encompass social needs in terms of long-term care and companions to ease loneliness [7,8].

The wide range of older people’s needs and their progressive functional decline increase their dependence on others, especially their family members and friends [5,9], such that their ability to maintain their health and improve their quality of life largely depends on family support (FS) [5].

Support, particularly by family members, has been recognized as an important predictor of psychological well-being among older people [10,11]. The types and sources of provision of support to older people vary in different countries. In Asian countries, family is the most important and sometimes only source of support [2,12,13]. The importance of FS for older people in the Iranian society is so high that their feeling of loneliness is often managed by living with their family members in their own homes [14]. In some countries such as the United States, Sweden, Germany, and United Kingdom, family may not be considered the main source of support for older people [15,16,17] because the responsibility of older people care are often granted to municipalities [18].

The amount and type of FS for older people also vary according to the immediate sociocultural context [19]. A comparative study on American and Chinese older people showed that sociocultural discrepancies influenced the importance of FS, so its effects on depression among Chinese older people were significantly greater than American people [20]. In other studies, family and friends have been recognized as the main sources of emotional support [21,22]. Conversely, children have been found to be the main source of financial support for their older parents in some countries [23,24]. Such differences are mainly due to differences in people’s attitudes towards old age, the meaning of support, and related measurement instruments in different contexts [11].

The main characteristic of the Iranian society is being “family-oriented”, which means that older members of the family are the sources of religious faith and love. In the Iranian culture and context, the provision of care to older people in their own homes by family members is considered an undeniable duty of families that also prevents older people’s transition into nursing homes. Given that paying tribute and respecting older people have been emphasized by the Iranian cultural and religious doctrine, Iranian families prefer to play the role of caregiver in their own homes to prevent violating the sense of sacred duty towards older family members [25,26].

Previous support-related studies on older people in Iran have often focused on the effects of social support on quality of life, loneliness, public health, and life satisfaction [12,27,28,29]. No study so far has explored FS for older parents in their own homes using qualitative research and from the perspectives of older people and their family caregivers in the Iranian context. Therefore, the present study aimed to remove this knowledge gap and to explore the main aspects of FS for older parents in home care.

## 2. Materials and Methods

### 2.1. Design

This qualitative study used a content analysis approach [30] and was conducted from August 2019 to August 2020. Participants were 21 older people recruited from parks and mosques where they gathered, mostly from two urban areas in Iran. Additionally, four family members, children of some participants, were included to improve the depth of data collection. Inclusion criteria were an age of over 65 years, their own homes as their residence, a willingness to sharing their experiences, and an absence of cognitive or psychological disorders. Sampling was purposively done with a maximum variation with regard to age, gender, education level, and socioeconomic status.

### 2.2. Data Collection

Face-to-face semi-structured interviews were held for data collection. Examples of the main interview questions were: “Can you explain about the types of help your children provide to you for shopping, cooking, fixing household appliances, cleaning house, and ensuring home safety?”, “Which types of help do you receive from your children for meeting household expenses, paying bills, and performing financial affairs?”, “What do your children do to understand your emotional status”, “What type of help do your children provide to you to prevent potential diseases and maintain your health?”, and “Will you explain about measures taken by your children to help you manage your problems with the use of technology?” Participants’ experiences were further explored during the interviews using probing questions. All interviews were held by the first author at participants’ preferred time and place. Additionally, the children of older people were asked in general about their roles in the support of their older parents, which helped to improve the depth of data collection. The length of the interviews was arranged according to participants’ willingness and was 20–45 min. All interviews were audio-recorded with the participants’ consent.

### 2.3. Data Analysis

Directed qualitative content analysis was used for data analysis [31]. This method improves the rigor of qualitative data analysis, makes the comparison of research findings possible, and yields practical results [32].

A literature search was performed to assess the current knowledge of family support for older people in the international literature, which led to the identification of predetermined categories [24,33,34,35]. Next, the interviews were transcribed word by word, and meaning units were identified and coded. According to their conceptual similarities, primary codes were grouped into these categories. Codes that could not be categorized into these categories were inductively grouped together to form new subcategories and categories [36].

### 2.4. Ethical Considerations

This study has the approval of the Ethics Committee of Tarbiat Modares University, Tehran, Iran (decree code: IR.MODARES.REC.1398.140). The participants received explanations about the aims of the study and the voluntariness of participation, and data confidentiality was ensured. They were also assured that only the main researcher and the study’s supervisor would have access to the raw interview data.

### 2.5. Trustworthiness

Trustworthiness was maintained through prolonged engagement with the data, member checking, and sampling with maximum variation. For member checking, a brief report of findings along with the interviews’ transcripts were provided to participants to confirm the congruence between their experiences and our findings. The process of data analysis was also supervised and confirmed by coauthors and external peers who were experienced in qualitative research and gerontology [37].

## 3. Results

A total of 25 people—14 older women, 7 older men, and 4 of their children—participated in this study. The mean of their age was 71 ± 4.8 years with a range of 66–85 years. Their education level ranged from illiterate to academic degree.

**Figure 1:**
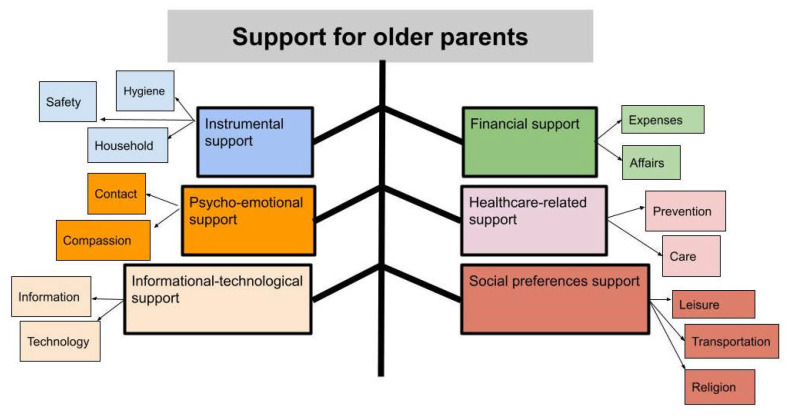
The aspects of family support for older parents in home care.

During the data analysis, 612 primary codes were generated and grouped into five main predetermined categories, namely “instrumental support”, “financial support”, ”psycho-emotional support”, “healthcare-related support”, “informational-technological support”, and one inductively developed category as “social preference support” (Figure 1). These six categories and subcategories were described using quotations of the participants.

### 3.1. Instrumental Support

The older people’s children provided them with different types of instrumental support consisting of support for personal hygiene and home cleaning, support for maintaining and fixing household appliances, support for structural safety, support for cooking, and support for washing clothes.

#### 3.1.1. Support for Personal Hygiene and Home Cleaning

Due to age-related physical decline, the older people were unable to independently perform personal hygiene and home cleaning tasks. Therefore, their children helped them keep personal hygiene such as bathing and nail doing on a weekly or monthly manner.

*I don’t have the physical ability that I had in my youth to clean my home. Therefore, my daughters and grandchildren alternately come and clean my home every other week or every month. Moreover, they bought a small vacuum cleaner by which I can clean around myself*.

(Participant 23; a 68-year-old woman)

#### 3.1.2. Support for Maintaining and Fixing Household Appliances

Most older people were unable to maintain and fix their household appliances; hence, their children assessed the appropriate function of their appliances and arranged fixing them.

*My children should come here before the onset of the cold and the warm seasons to assess and prepare warmers or cooler. My older son performs simple fixings. For example, if there is a stuck-out nail in the furniture, which tears clothes, my son brings a hammer and fixes it. Or, if he sees that the water faucet is dripping, he fixes it*.

(Participant 7; a 72-year-old woman)

#### 3.1.3. Support for Structural Safety

The participants’ experiences showed that their home designs were not age-appropriate, so they had concerns about the risk of fall. Therefore, their children provided them with some kinds of support in order to ensure their structural safety and reduce their concerns. For example, they installed toilet seat in the rest room, grab bars in the bathroom, and handrails in the staircase.

*Nowadays, homes have not been designed to be appropriate for older people. I had to take extreme care in order not to slip on floor ceramic tiles, because fractures in older people do not heal. I have slipped several times so far. Thanks God! I just experienced a wrist sprain and now, my wrist aches when it gets cold. Previously, homes were not like this and floor was covered with mosaic tiles. My children have bought me slippers in order not to slip and have installed grab bars in the rest room and the bathroom. They have also covered the kitchen floor with moquette*.

(Participant 8; a 79-year-old man)

#### 3.1.4. Support for Cooking

The participants lived alone, and they were in no mood for cooking for themselves. Therefore, their children cooked several meals for them or provided them with the necessary conditions for cooking simple foods.

*I can make simple foods. Of course, my younger daughter prepares and packs necessary food stuffs for me. For example, she cuts and packs meat for each meal and brings me fried onion so that I don’t need to stand on the feet for long time for cooking. My daughter-in-law also makes appropriate foods for me as she knows that I have no teeth*.

(Participant 4; a 80-year-old woman)

#### 3.1.5. Support for Cloth Washing

Inability to wash their clothes due to age-related physical decline or inability to use washing machine due to age-related memory impairment was reported by the older people. Therefore, their children, particularly their daughters, returned to their homes on a weekly or monthly basis in order to help them wash their clothes.

*My hands are not strong enough anymore to wash clothes and, hence, I use washing machine. I put dirty clothes in a basket and my daughters come here at the weekend, wash them with the machine, and hang them from the clothesline*.

(Participant 19; a 67-year-old woman)

### 3.2. Financial Support

The older people’s children provided them with direct or indirect financial support in terms of household expenses and support for doing financial affairs.

#### 3.2.1. Support for Household Expenses

Though the older people’s their income reduced in the late adulthood, their expenses increased due to age-related health problems and family enlargement. Consequently, their children provided them with financial support by paying for some of their expenses.

*My income is inadequate. Although we [have a private house] don’t pay rent, we can’t afford all expenses. I have a single boy whose expenses are with me. My wife also has knee pain and receives physiotherapy, while insurance companies don’t cover all treatment-related costs. I’m also sick and have healthcare-related costs. However, my daughter financially helps me though she is married and doesn’t live with us*.

(Participant 1; a 69-year-old man)

#### 3.2.2. Support for Doing Financial Affairs

Despite great life experiences, the older people could not effectively perform their financial affairs due to many different reasons such as age-related memory impairment, limited literacy, and the dynamicity of banking activities. Therefore, their children provided them with complete or partial support for doing their financial affairs such as cash withdrawal and financial calculations.

*I have memory impairment. My children perform the affairs related to my installments so that I can decide for my other expenses*.

(Participant 15; a 68-year-old woman)

### 3.3. Psycho-Emotional Support

The older people’s children provided them with psycho-emotional support through frequently contacting them and showing compassion towards them. They noted that their children’s psycho-emotional support improved their mental status and mood, as well as relatively reducing their age-related psychological strains.

#### 3.3.1. Frequent Greeting Contacts

One of the good behaviors of their children was their frequent face-to-face or telephone contacts for greeting made either daily or weekly. They did not put great importance to the time of the contacts but considered them indicative of their importance for their children and their children’s respect for them.

*I’ve become old. What do we need except for happiness? I like my sons and daughters-in-law to pay more attention to me and visit me every two to three days and contact me every day. My children’s support and attention are very valuable and make me happy*.

(Participant 20; a 68-year-old woman)

#### 3.3.2. Showing Compassion

The older people’s children were also compassionate to them, felt responsibility towards them, and willingly attempted to fulfill their needs before their request.

*When I had a work or some home appliance had been out of work, they knew what to do and how to help. For example, when I had a party, they came and performed shopping. When I wanted to move something at home, they willingly did it for me*.

(Participant 9; a 72-year-old man)

### 3.4. Healthcare-Related Support

This type of support was provided by the children of older people by taking preventive measures and giving them care services. It protected the older people’s health, maintained their autonomy, and improved their quality of life.

#### 3.4.1. Preventive Measures

Measures taken by their children to prevent potential health problems in the older people included encouragement for engagement in self-care activities, careful attention to dietary regimen, and periodical medical examinations.

*My children frequently recommend me what to eat and not to eat. For example, they don’t put saltshaker at the table, remind me that salt and fat are not good to my hypertension, and warn me against excessive use of salt and sugar. They also recommend me to take fruits. They are very sensitive to my eating*.

(Participant 7; a 71-year-old man)

#### 3.4.2. Care Measures

The older people suffered from at least one chronic illness and took multiple medications. Therefore, healthcare-related support by their children encompassed reminding them to take their medications at appropriate times, encouraging them to adhere to their treatments, and buying them their medications. Moreover, the children of participants with low literacy skills dispensed their medications for each 24-h cycle.

*I don’t know how to take my medications. My daughter has bought me a pill organizer and puts my morning, noon, and evening medications in it. Then, I can easily take them*.

(Participant 23; an 85-year-old woman)

Moreover, despite their own resistance and unwillingness, their children encouraged and convinced them to have periodical medical visits.

*I like to use self-treatment for my health problems such as common cold. However, my children disagree with me mostly and take me to the doctor or oblige me to go to the doctor*.

(Participant 15; a 68-year-old woman)

### 3.5. Informational-Technological Support

The older people’s children informed them of news, provided them with education about how to use electronic devices, and simplified the use of electronic devices using paintings or color labels. Their children’s informational-technological support had a significant role in improving their quality of life.

#### 3.5.1. Informational Support

Their children provided them with different information about daily events in society and considered such informational support effective in preventing potential financial and physical health problems.

*My daughter and son-in-law have frequently emphasized that I should see the face of those who ring my home bell through the video door-phone. My son-in-law tells me about cheatings and asks me not to disclose personal information over telephone*.

(Participant 25; a 76-year-old woman)

#### 3.5.2. Technological Support

The complexity and dynamicity of electronic devices, as well as the reduced learning abilities of the participants, have caused the older people to have no interest in their use. Their children provided them with continuous education about these devices and encouraged them to use them.

*My children have bought me a new mobile phone; but I’m illiterate and can’t use it. My children have put their photos in the contact list of the phone, and I can easily contact them by taping their photos*.

(Participant 18; a 69-year-old woman)

*My daughter washes my clothes using washing machine because I can’t use it. She has trained me how to use the machine for several times, but I cannot learn it. Of course, I could use my previous washing machine, but can’t use this new one*.

(Participant 22; a 80-year-old man)

### 3.6. Social Preference Support

A willingness to engage in social activities together with others and their family members was mentioned by the older people. Their children supported them to engage in their preferred social activities including leisure, transportation, and religious affairs.

#### 3.6.1. Support for Leisure Activities

Their children considered activities for their leisure time and helped them perform their favorite leisure activities.

*My children bought me two puzzles to entertain me. I do them many times. It is good for my memory. I also like painting. One of my grandchildren brought me several drawing notebooks and color pencils and told me, ‘Grandmother! Do drawing to pass your time.’ They also have put a chair in a safe place in the balcony for me to seat and watch people and children in the park*.

(Participant 4; a 80-year-old woman)

#### 3.6.2. Support for Transportation

Their children provided them with adequate transportation support for visiting their favorite places and other family members, travelling, shopping, and visiting the doctor. Thereby, opportunities to get more engaged in social activities were provided.

*My children take me on trips since they have private car and I’m comfortable with them. They take me wherever I want to go such as to doctor, hospital, and parties. I even perform heavy shopping with their help*.

(Participant 7, a 71-year-old man)

#### 3.6.3. Support for Religious Affairs

The participants discussed getting involved in religious affairs and noted that their children supported them to perform religious affairs and participate in religious family rituals.

*Each year, I hold a religious ritual at home and invite friends and relatives. Now, I can’t do such things due to pain in my limbs. However, my children always help me hold the ritual at my home and do all things from the beginning to the end*.

(Participant 14; a 67-year-old woman)

*My children are attentive to anniversaries. For example, they come here in the Yalda night, Mother’s Day, and Father’s Day. I’m very happy when they are here*.

(Participant 17; a 66-year-old woman)

## 4. Discussion

This study explored the main aspects of FS for older parents in home care from the perspectives of older people and their family caregivers. The exploration of caregiving experiences helps with the development of insights regarding the aging process and the understanding of how humanistic interactions and personhood can be incorporated into care initiatives [38].

According to this qualitative research, the main aspects of FS were grouped into five predetermined categories and one new category. They were “instrumental support”, “financial support”, “psycho-emotional support”, “healthcare-related support”, “informational-technological support”, and “social preference support”.

Instrumental support included support for performing daily living activities. Due to the old age, the older people needed and received their children’s support for washing their clothes, cooking, and use of technology. Older people with a higher educational level and better financial status may have less dependence on their children because they can use private companies’ services for home cleaning and daily shopping, though they expect their children to pay more attention to their needs. In stressful situations during health problems, in order to maintain a sense of competence, instrumental support helps older people fulfill personal needs such as for shopping, cooking, and home cleaning [39]. Instrumental support has been shown to have a positive relationship with psychological well-being [11] and a negative relationship with depression [39]. Most older people suffer from psychological problems and therefore need specific support for managing their daily life activities and self-care [6]. A study reported that one fifth of healthy older parents needed instrumental support [39]. Older people above seventy years have a growing dependence for conducting their activities of daily living, particularly for those activities that involve lower limbs. On the other hand, they can independently perform activities that involve the upper limbs such as manual tasks, eating, and position changing until the age of eighty years [40].

A greater dependence among older men whose wives had died was observed. Instrumental support is of greater importance for older men and influences their life satisfaction, general health, and sense of loneliness [29,41]. However, older men often preferer to receive instrumental support from their wives [42]. Older men who live with their wives have limited dependence on their children, and their wives’ support is more beneficial to them than peer support [43]. However, another study indicated that older people’s dependence was not gender-bound [40]. The growing prevalence of successful aging means that older people are becoming increasingly independent in performing their daily life activities and fulfilling their personal needs [44]. Though the number of independent older people aged 65–74 years has dramatically increased, growing independence does not necessarily mean that they do not need support and care, because they usually have complex care needs that should be fulfilled by healthcare services and social care systems [17].

Structural support was another main aspect of instrumental support in the present study. The need for children’s support for fall prevention was highlighted. Falls are a highly prevalent problem among older people due to slipping, poor lighting [45], and age-inappropriate home design [46]. Therefore, measures should be taken to ensure safety in older people’s homes [47].

Financial support was the second most important aspect of FS for the older parents in this study. Retirement is associated with a decreased income, but aging is associated with increased healthcare costs due to age-related chronic conditions. Children provided financial support to their older parents if they had inadequate income or faced financial problems. The financial security of older people has turned into a major sociopolitical issue [48], and older people often report financial strains due to their inadequate income, inability to fulfill their children’s financial needs, high healthcare costs, inability to give gifts to others, and limited perceived social and financial support [49,50]. After retirement, older people have three main sources of income, namely personal assets and savings, transfers by children, and public transfers supported by people who pay taxes. Public transfers are the main sources of financial support for older people in most developed countries, though family transfers are the most important source of financial support for older people in transitional countries [51]. On the other hand, the responsibility of financial support for older people in some cultures is with their children, such that older people expect their adult children to financially support them, particularly when they have inadequate income to fulfill their needs [23,52].

The third most important category of FS for older parents was psych-emotional support. It meant that older people from both genders were interested in family parties, frequent visits by children, and daily greeting contact. Emotional support for older people consists of affective interactions between older people and their significant others, which comprise the most valuable type of support from other people’s perspectives [12]. Emotional support by family members improves psychological wellbeing [11] and happiness [53] among older people. Nonetheless, some family members, particularly adult children, may greatly focus on instrumental and financial support for their older parents at the expense of emotional support [54]. Therefore, they should be provided with education about the importance of emotional support for older parents in order to improve their sense of satisfaction and wellbeing.

Healthcare-related support was another major aspect of FS reported in this research. The National Academies of Sciences, Engineering, and Medicine also considers family members as the main source of support for those people who have short- or long-term care-related needs [34]. Family is also a main source of healthcare-related support in the Iranian context. With aging, older people experience different physical health problems such as visual impairments, arthralgia, respiratory disorders, diabetes mellitus, depression, and dementia [1,55]. Some of these problems are due to unhealthy lifestyle behaviors such as immobility, obesity, and inappropriate eating habits [55]. Therefore, health-promoting behaviors and healthy lifestyles are important strategies for health maintenance and promotion [56]. Lifestyle modifications for older people can be performed by providing them with strong educational, counseling, and financial support [57]. Older people need comprehensive information in order to successfully modify their lifestyle, closely adhere to their medications and healthy lifestyle behaviors, and actively collaborate with healthcare providers [58,59]. Therefore, healthcare providers can facilitate lifestyle modifications and healthy aging among older people by developing comprehensive self-care programs [55] and providing them and their family members with simple quality educations about structural safety, nutritional needs, physical activity, and common age-related disorders [60,61].

Support can be provided by a wide range of sources such as friends, family, neighbors, and other people in society [62]. It is noteworthy that the healthcare-related needs of older people are context-bound and affected by different factors such as the immediate sociocultural context [63]. In our study, the older parents received healthcare-related support from their spouses and daughters. In Iran, female family caregivers have greater acceptance due to dominant traditions and cultural beliefs in the country [64]. The greater involvement of female family members in caregiving to older people is common both in Eastern and Western cultures [34,59,65,66]. It has been stated that female family caregivers spend more time in the provision of personal-care tasks than male caregivers. Therefore, they experience a greater burden of care because of the greater physical strain and psychological distress [67]. That burden of care can be accompanied by negative physical and psychosocial consequences on their health and quality of life [68,69,70], as well as lead to the mistreatment [71], poor quality of care, neglect, and even institutionalization of older people in long-term healthcare settings [70,72]. Therefore, healthcare professionals in community healthcare settings should support family caregivers and strengthen the feelings of coherence and attachment between older people and their caregivers in order to reduce the burden of care [73]. 

Informational-technological support was an aspect of FS for the older parents. They could not personally use electronic devices such as computers or mobile phones, so they needed help and support. Nowadays, there are different electronic devices in homes, some of which are sophisticated and difficult to use, but they are useful to older people because they make their lives easier and provide them with the opportunity to have more social interactions. Consequently, older people need to receive education about the effective and safe use of these devices [74]. Older people may have both positive attitudes and worries regarding the use of electronic devices such as mobile phones [75,76,77]. Consequently, only a small proportion of older people use electronic devices and services. A study in Iran showed that only 10% of people aged above fifty years used a personal computer, laptop, or tablet, and only 18% of them used electronic banking services [78]. Many different factors could contribute to the limited use of technology by older people. The barriers have been shown to be age-related, including physical and mental problems, and personal, such as low literacy skills, limited access to technology, negative attitudes towards technology, technological barriers [59], limited interest in technology, and unfamiliarity with technology [78]. Moreover, some older people are unable to use technology because electronic devices are not age-appropriate and their user manuals are complex and difficult to understand [59]. All these findings highlight the necessity of taking age-related considerations into account when designing electronic devices for older people [79]. Family members, particularly grandchildren, also need to provide their older parents with simple and quality education about the use of technology [80,81].

Social preference support, as another aspect of FS, was found to facilitate the older people’s engagement in their preferred social activities. The favorite social activities of older people in Iran are to walk and to follow religious rituals in mosques, where they can find new friends, acquire a sense of spiritual peace, and interact with others [82]. However, some older people in other contexts may be mostly interested in physical activities and gardening [83]. Older people’s engagement in social activities and interactions is affected by different personal, environmental, and social factors and gives them a sense of satisfaction [84], happiness, social belongingness [85], family attention, and family interest [86]. Additionally, it improves their physical, mental, and cognitive health, thereby helping them experience a successful aging [87]. It also has positive effects on intergeneration relationships, young people, families, and societies [88]. Therefore, family members should encourage older people and support them to actively participate in social activities. The expansion of social networks has been shown to prevent the sense of loneliness and promote perceived social support in older adults [89].

The strength of this study lies in the data collection from both older people and their family members in two urban areas of Iran. Similarities between our study findings and those of other studies in other contexts were shown in the discussion section, which indicated the transferability of our findings to other similar contexts. Age-related physical and mental health problems might have affected the older people’s ability to fully participate in the data collection and share their experiences. However, the researcher used all rapport initiatives in the communication process and spent enough time with them to reduce any of these impacts on the quality of data collection. More studies with larger samples sizes and in other cultures are required to improve our understanding of this phenomenon and help to improve the transferability of our research findings.

## 5. Conclusions

According to this study findings, six main aspects of FS for older parents in home care were instrumental support, financial support, psycho-emotional support, healthcare-related support, informational-technological support, and social preference support. Our study findings can be used to strengthen FS for older people, thereby improving their quality of life and satisfaction with life in home care. The holistic approach of older people care indicates the necessity of the consideration of older people’s physical, psychological, social, and spiritual needs during the development of strategies that aim to improve the quality and safety of home care. Our findings have implications for improvements of the quality of home care and reductions of the feeling of loneliness among older people. Community healthcare providers should involve family caregivers in the process of home care and enhance their active role in the improvement of older people’s well-being and health.

More studies using the quantitative design should be carried out to examine the effects of the provision of various types of support by family caregivers on older people’s wellbeing and quality of life.

## Data Availability

Restrictions apply to the availability of data from this research because of anonymity of the participants and confidentiality matters.
